# *In Vivo* and* In Vitro* Genotoxic and Epigenetic Effects of Two Types of Cola Beverages and Caffeine: A Multiassay Approach

**DOI:** 10.1155/2016/7574843

**Published:** 2016-07-04

**Authors:** Marcos Mateo-Fernández, Tania Merinas-Amo, Miguel Moreno-Millán, Ángeles Alonso-Moraga, Sebastián Demyda-Peyrás

**Affiliations:** ^1^Department of Genetics, University of Córdoba, Rabanales Campus, 14071 Córdoba, Spain; ^2^Institute of Veterinary Genetics (IGEVET), Facultad de Ciencias Veterinarias, UNLP-CONICET, Universidad Nacional de La Plata, 1900 La Plata, Argentina

## Abstract

The aim of this work was to assess the biological and food safety of two different beverages: Classic Coca Cola*™* (CCC) and Caffeine-Free Coca Cola (CFCC). To this end, we determined the genotoxicological and biological effects of different doses of lyophilised CCC and CFCC and Caffeine (CAF), the main distinctive constituent. Their toxic/antitoxic, genotoxic/antigenotoxic, and chronic toxicity (lifespan assay) effects were determined* in vivo* using the* Drosophila* model. Their cytotoxic activities were determined using the HL-60* in vitro* cancer model. In addition, clastogenic DNA toxicity was measured using internucleosomal fragmentation and SCGE assays. Their epigenetic effects were assessed on the HL-60 methylation status using some repetitive elements. The experimental results showed a slight chemopreventive effect of the two cola beverages against HL-60 leukaemia cells, probably mediated by nonapoptotic mechanisms. Finally, CCC and CAF induced a global genome hypomethylation evaluated in LINE-1 and Alu M1 repetitive elements. Overall, we demonstrated for the first time the safety of this famous beverage in* in vivo* and* in vitro* models.

## 1. Introduction

Diet may modify cancer risk and tumor behavior since nongenotoxicological modulation as epigenetic regulatory processes may be susceptible to changes caused by environmental factors. Therefore, constituents in food and dietary supplements could be involved in changes in the gene expression, increasing the risk of developing some type of cancer all over the life inducing epigenetic changes [1, 2]. Genotoxicological screening tests have been extensively used over time for assessing the health properties of compounds prior to being considered as safe substances. Nowadays, the list of foods with documented health-benefit activities is endless, and scientific evidence supporting the concept of health-promoting food ingredients is steadily growing [3].

Originally developed as medical supplements, cola-based drinks and several beverages such as beer and wine were proposed as medicinal substances [4, 5]. However, a relationship between the consumption of these beverages and an increase in the prevalence of several diseases such as child obesity, diabetes, hypertension, and dental diseases was also demonstrated [6–8]. In spite of the worldwide importance and spread of cola beverages, studies assessing their effects on health and wellbeing are quite scarce [9]. On the contrary, caffeine (CAF), which is a key ingredient in cola beverages as well as in coffee, tea, and some medicines, is one of the most investigated substances, probably due to the lack of consistent results over time [10–12]. In* D. melanogaster*, CAF has been related to a positive lifespan increase [13], but the results were contradictory when apoptotic and DNA-programmed fragmentation effects were studied [14, 15].

*Drosophila* is being used more frequently as a model for many human diseases, including cancer [16–18]. Reiter et al. [19] determined that 77% of human disease genes are conserved in this fly, making it an important preliminary model in the study of human diseases. These flies are also used often to determine the mutagenicity of some substances. Somatic cell mutations and apoptosis-resistance, widely associated with genetic toxicity and carcinogenicity, are frequently assayed using the* in vivo Drosophila melanogaster* model through the Somatic Mutation and Recombination Test (SMART) [20, 21], which was demonstrated as a reliable assay to detect genotoxic and antigenotoxic activity of single compounds and complex mixtures [22, 23]. More recently, this fly model was also increasingly used to study life extension since there is a high homology between invertebrate and human genes involved in aging process [24, 25]. On the other hand, the determination of cytotoxicity, DNA internucleosomal fragmentation, and DNA single/double strand breaks in HL-60 promyelocytic cells is also used as a first step to detect toxicity, necrosis, and apoptosis in chemoprevention processes [26–28].

Biomedical research is focused on modifying the methylation pattern as a tool to understand cancer processes and other diseases. Medical epigenetic might take part in the junction between the genome and the environment, to modulate the effects of deleterious genes [29, 30].

Therefore, the aim of this study was to determine the potential toxicity and DNA protecting capabilities of lyophilised CCC, lyophilised CFCC, and CAF. Several end-points related to degenerative processes, including toxicity, antitoxicity, genotoxicity, antigenotoxicity, and longevity were determined using an* in vivo Drosophila* model. Furthermore,* in vitro* chemopreventive activity of these compounds was also determined by assessing their cytotoxicity and DNA damage capability producing internucleosomal fragmentation or strand breaks in an HL-60 promyelocytic human cancer model as well as the modulation of its methylation status in genomic repetitive sequences.

## 2. Materials and Methods

### 2.1. Samples

Two coke beverages, CCC and CFCC, and one of their principal compounds, CAF (1,3,7-trimethylpurine-2,6-dione), were assayed. Drinks were bought at a local market (Córdoba, Spain), lyophilised (SCAI, University of Córdoba), and stored at room temperature in a dark and dry atmosphere until use. CAF was obtained from ACROS (108.160100).

The analysis of CAF content was performed by HPLC/DAD (Perkin Elmer) in reverse phase (column C-18, 150 × 2.1 mm), with a gradient of water/phosphoric buffer and methanol as mobile phase at a 1 mL/min flow rate. The injection volume was 10 *μ*L and the column temperature at 45°C. The CAF identification was performed by retention time and spectrum adjustment obtained by DAD (SCAI, University of Córdoba).

### 2.2. *In Vivo* Fly Stocks

Two* Drosophila melanogaster* strains with genetic markers that affect the wing-hair phenotype were used: (i)* mwh/mwh*, carrying the recessive mutation* mwh* (multiple wing hairs) [31] and (ii)* flr* ^*3*^ */In (3LR) TM3, rip* ^*p*^ *sep bx* ^*34e*^ *e* ^*s*^ *Bd* ^*S*^, where* flr* ^*3*^ (*flare*) [32] marker is a homozygous recessive lethal mutation which is viable in homozygous somatic cells once larvae start developing and produce deformed trichomonas.

### 2.3. *In Vitro* Cell Culture Conditions

Promyelocytic human leukaemia (HL-60) cells were grown in RPMI-1640 medium (Sigma, R5886) supplemented with heat-inactivated foetal bovine serum (Linus, S01805), L-glutamine 200 mM (Sigma, G7513), and 1x antibiotic-antimycotic solution (Sigma, A5955). Cells were incubated at 37°C in a humidified atmosphere of 5% CO_2_. Cultures were plated at 2.5 × 10^4^ cells/mL density in 10 mL culture bottles and passed every 2 days.

### 2.4. *In Vivo* Assays

#### 2.4.1. Toxicity and Antitoxicity Assays

Toxicity was assayed according to our standard protocols. Both lyophilised beverages (CCC and CFCC) were tested at five concentrations: 0.7, 3, 6, 25, and 100 mg/mL. The same number of CAF concentrations (0.04 mM, 0.016 mM, 0.032 mM, 0.128 mM, and 0.51 mM) was also tested according to quantity declared by Chou and Bell [33] and HPLC results obtained in the present study (75.544 mg/L). Negative (H_2_O) and positive (0.15 M H_2_O_2_) toxicant concurrent controls were also assayed. Test groups consisted of larvae fed with* Drosophila* Instant Medium (Formula 4–24, Carolina Biological Supply, Burlington, NC) supplemented with the beverage concentrations tested. Emerging adults of all groups were counted and toxicity was determined as the percentage of hatched individuals in each treatment compared with the negative control. Antitoxicity was assessed using the same procedure and experimental concentrations as in toxicity assays, but in combined treatments with 0.15 M H_2_O_2_ and comparing the percentage of emerging adults with the positive toxicant control [34]. Chi-square test was used to determine if the tested compounds significantly inhibited the survival of flies. Negative control values were considered as those expected in Chi-square formula used in toxicity assay and positive control values in antitoxicity assays [35]. The same concentrations of toxicity and antitoxicity assays within the same substance were also compared.

#### 2.4.2. Genotoxicity and Antigenotoxicity Assays

Genotoxicity assays were carried out following the wing spot test standard procedure [20]. Briefly, transheterozygous larvae for* mwh* and* flr* ^*3*^ genes were obtained by crossing four-day-old virgin* flr* ^*3*^ females with* mwh* males in a 2 : 1 ratio. Four days after fertilization, females were allowed to lay eggs in fresh yeast medium (25 g yeast and 4 mL sterile distilled water) for 8 h in order to obtain synchronised larvae. After 72 h, larvae were collected, washed with distilled water, and clustered in groups of 100 individuals. Each group was fed with a mixture containing 0.85 g* Drosophila* Instant Medium (Formula 4–24, Carolina Biological Supply, Burlington, NC) and 4 mL water supplemented with the tested compounds at fixed concentrations (the highest and second lowest from the toxicity assays) and negative (H_2_O) and positive (0.15 M H_2_O_2_) controls until pupae hatching (10–12 days). Adult flies were collected and stored in 70% ethanol until the wings were removed and mounted on slides using Faure's solution. Mutant spots were assessed in both dorsal and ventral surfaces of the wings in a bright light microscope at 400x magnification. The frequencies of each type of mutant clone per wing (single, large, or twin spot) were compared to the concurrent negative control and analysed applying the binomial Kastenbaum and Bowman Test [36]. Antigenotoxicity tests were performed following the method described by Anter et al. [37]. The same compounds and concentrations were assayed in combined treatment with hydrogen peroxide (0.15 M) acting as concurrent genotoxicant. Single and twin spots per wing were also recorded and compared with the concurrent positive control as described before. The recombination percentage was calculated following Valadares et al. [38] procedure and the inhibition percentages (IP) for the combined treatments were calculated from the control-corrected frequencies of clone formation per 10^5^ cells, according to Abraham [39]: IP = [(genotoxin alone − combined treatment)/genotoxin alone] × 100.

#### 2.4.3. Chronic Treatments: Lifespan and Healthspan Assays

In order to obtain comparable results in all the* in vivo* assays, we used an F_1_ progeny from* mwh* and* flr* ^*3*^ parental strains produced by 24 h egg-laying in yeast for all the longevity trials. We also tested the same compounds and concentrations as in the toxicity/antitoxicity experiments. Lifespan assays were carried out at 25°C according to the procedure described by Fernandez-Bedmar et al. [23]. Briefly, synchronised 72 ± 12-hour-old transheterozygous larvae were washed in distilled water, collected, and transferred in groups of 100 individuals into test vials containing 0.85 g* Drosophila* Instant Medium and 4 mL of the different concentrations of the compounds to be assayed. Emerged adults from pupae were collected under CO_2_ anaesthesia and placed in groups of 25 individuals of the same sex into sterile vials containing 0.21 g* Drosophila* Instant Medium and 1 mL of different concentrations of the compounds to be tested. Flies were chronically treated during all their life. The number of survivors was determined twice a week.

### 2.5. *In Vitro* Assays

#### 2.5.1. Cytotoxicity Assay

The effect of the assayed compounds on cell viability was determined by the trypan blue exclusion test according to our standard procedures [37]. HL-60 cells were placed in 96-well plates (2 × 10^4^ cells/mL) and cultured for 72 h and supplemented with the same concentrations of CCC, CFCC, and CAF from our toxicity/antitoxicity assays. The wide range of tested concentrations was intended to estimate the cytotoxic inhibitory concentration 50 (IC_50_). After culture, cells were stained with a 1 : 1 volume ratio of trypan blue dye (Sigma, T8154) and counted in a Neubauer chamber at 100x magnification. The survival percentage of each treatment compared with the control was recorded in three independent replicates.

#### 2.5.2. DNA Fragmentation Status

The ability of our compounds to induce DNA fragmentation was determined as described by Anter et al. [40]. Briefly, 10^6^ HL-60 cells were cocultured with 5 different concentrations of CCC, CFCC, and CAF (as selected in the toxicity/antitoxicity assays) for 5 h. After treatment, genomic DNA was extracted using a commercial kit (Blood Genomic DNA Extraction Mini Spin Kit, Canvax Biotech, Cordoba, Spain). Subsequently, DNA was incubated overnight with RNase at 37°C and quantified in a spectrophotometer (Nanodrop® ND-1000). Finally, 1200 ng DNA was electrophoresed in a 2% agarose gel for 120 min at 50 V, stained with ethidium bromide, and visualised under UV light. The apoptosis process is recognised by the appearance of internucleosomal DNA fragments that are multiple of 200 base pairs.

#### 2.5.3. Clastogenicity: SCGE (Comet Assay)

DNA integrity was assayed by SCGE as described by Olive and Banáth [41] with minor modifications. HL-60 cells (5 × 10^5^) in exponential growing phase were incubated in 1.5 mL of culture medium supplemented with different CCC, CFCC (0.7, 6, and 25 mg/mL), and CAF (0.004, 0.032, and 0.51 mM) concentrations for 5 h. After treatment, cells were washed twice and adjusted to 6.25 × 10^5^ cells/mL in PBS. Electrophoresis gels were prepared pouring a 1 : 4 dilution (cells in liquid low-melting-point agarose at 40°C, A4018, Sigma) into slides. Gels were covered with a coverslip and allowed to solidify at RT for 30 min. Once the slides solidified, the coverslips were carefully removed and slides were bathed in freshly prepared lysing solution (2.5 M NaCl, 100 mM Na-EDTA, 10 mM Tris, 250 mM NaOH, 10% DMSO, and 1% Triton X-100; pH 13) for 1 h at 4°C. Thereafter, slides were equilibrated in alkaline electrophoresis buffer (300 mM NaOH and 1 mM Na-EDTA, pH 13) for 20–30 min at 4°C. Once equilibrated, the slides underwent electrophoresis (20 V, 400 mA for 15 min) in the dark and were immediately neutralised in cold neutral solution (0.4 M Tris-HCl buffer, pH 7.5) for 10 min. Finally, slides were dried overnight at RT in the dark. Gels were stained with 7 *μ*L propidium iodide and photographed in a Leica DM2500 microscope at 400x magnification. At least 100 single cells from each treatment were analysed using the Open Comet*™* software [42]. The Tail Moment (TM) data were analysed applying a one-way ANOVA and* post hoc* Tukey's test with SPSS Statistics for Windows, Version 19.0 (IBM 2010), to determine the effect of the tested compounds on HL-60 cell DNA integrity.

#### 2.5.4. Methylation Status of HL-60 Cells

HL-60 cells were treated with different concentrations of CCC (3 mg/mL and 100 mg/mL), CFCC (3 mg/mL and 100 mg/mL), and CAF (0.016 mM and 0.51 mM) for 5 hour. Then, DNA was extracted similarly to previously described DNA fragmentation assay. After that, the DNA was converted with bisulphite (EZ DNA Methylation-Gold*™* Kit). Bisulphite-modified DNA was used for fluorescence-based real-time quantitative Methylation-Specific PCR (qMSP) using 5 *μ*M of each forward and reverse primer (Isogen Life Science BV), 2 *μ*L of iTaq*™* Universal SYBR® Green Supermix (Bio-Rad, it contains antibody-mediated hot-start iTaq DNA polymerase, dNTPs, MgCl2, SYBR Green I Dye, enhancers, stabilizers, and a blend of passive reference dyes including ROX and fluorescein) and 25 ng of bisulphite converted genomic DNA.

PCR conditions included initial denaturalisation at 95°C for 3 minutes and amplification which consisted of 45 cycles at 95°C for 10 seconds, 60°C for 15 seconds, and 72°C for 15 seconds, taking picture at the end of each elongation cycle. After that, melting curve was determined increasing 0.5°C each 0.05 seconds from 60°C to 95°C and taking pictures.

QMSP was carried out in 48-well plates in MiniOpticon Real-Time PCR System (MJ Mini Personal Thermal Cycler, Bio-Rad) and were analysed by Bio-Rad CFX Manager 3.1 software. The housekeeping Alu-C4 was used as a reference to correct for total DNA input. Alu-C4 and the target repetitive elements Alu M1, LINE-1, and Sat-*α* were obtained from Isogen Life Science and their sequences are shown in Table 1. Each sample was analysed in triplicate.

The results of each C_T_ were obtained from each qMSP. Data were normalised with the housekeeping Alu C4 using the Nikolaidis et al. [45] and Liloglou et al. [46] comparative C_T_ method (ΔΔC_T_). One-way ANOVA and* post hoc* Tukey's test are used to evaluate the differences between the tested compounds, repetitive elements, and concentrations.

## 3. Results

### 3.1. *In Vivo* Assays

#### 3.1.1. Toxicity/Antitoxicity

Toxicity assays showed that CCC, CFCC, and CAF are not toxic to* D. melanogaster* larvae (Table 2, simple treatment).

CFCC was significantly toxic only at the highest concentration. All the studies and results on CAF must be viewed with caution, since CAF shows a dose-dependent effect and it is known to be toxic at high concentrations [47].

Antitoxicity results showed that CCC and CFCC exerted an overall significant protective effect against H_2_O_2_-induced toxicity in* Drosophila* larvae, at most of the tested concentrations, with a negative dose-dependent effect (Table 2, combined treatment). Although CCC and CFCC were able to revert in some extent the damage caused by hydrogen peroxide, the survival obtained in antitoxicity assay was lower than toxicity assay in flies treated with 6, 25, and 100 mg/mL of these beverages. On the other hand, the 2 lowest concentrations were able to totally revert the oxidative damage caused by the used genotoxin. On the contrary, none of the assayed CAF concentrations produced any significant protective effect.

#### 3.1.2. Genotoxicity/Antigenotoxicity

Table 3 shows the results obtained in the genotoxicity assays (SMART). After applying binomial Kastenbaum-Bowman Test, all tested substances were nongenotoxic with negative results.

Hydrogen peroxide is a potent inducer of oxidative damage and mediator of ageing [48]. It has been used as a genotoxicant in many assays using* Drosophila* as an experimental animal [23, 40] as well as in other models. The mutation rates obtained in our study for this genotoxin (0.438 clones/wing) fall into the usual range described by different laboratories, validating the accuracy of the geno/antigenotoxicity assays.

One of the important characteristics of the SMART is that it allows quantification of the different types of DNA damages induced by genotoxic compounds (recombination versus mutation). In the balancer-heterozygous genotype (*mwh*/TM3, Bd^S^)* mwh* spots are produced predominantly by somatic point mutation and chromosome aberrations. By scoring* mwh*/TM3 balancers-heterozygous wings it is possible to quantify the recombinogenic potency of the positive control. The frequency of* mwh* clones on the marker transheterozygous wings (*mwh* single spots plus twin spots) was compared with the frequency of* mwh* spots on the balancer transheterozygous wings. The difference in* mwh* clone frequency is a direct measure of the proportion of recombination. A total mutation rate of 0.2 in the* mwh*/TM3 wings has been obtained and when it is compared to the mutation rate of the marker wings (0.438) thus 54% [1 − (0.819/1.795) × 100] of the genotoxic events induced by H_2_O_2_ are due to recombinogenicity.

Antigenotoxicity results indicated that CCC, CFCC, and CAF could desmutagenise the genotoxic effect of H_2_O_2_, except for the highest tested concentration of CAF. CCC was the most antigenotoxic tested compound (IP: 166.67% and 98.88% for 3.125 and 100 mg/mL, resp.). CFCC IP was 96.93% and 121.76% for similar CCC concentrations and the 0.016 mM CAF IP was 142.96%. All the clone frequencies in combined treatment were compared to the positive control H_2_O_2_.

Recombinogenicity values for combined treatments ranged between 55 and 89%, where these figures are higher than their respective recombinogenicity induced by the positive control (54%). Therefore, our compounds induced antimutagenic activity rather than antirecombinogenic activity.

#### 3.1.3. Chronic Treatment

**Figure 1:**
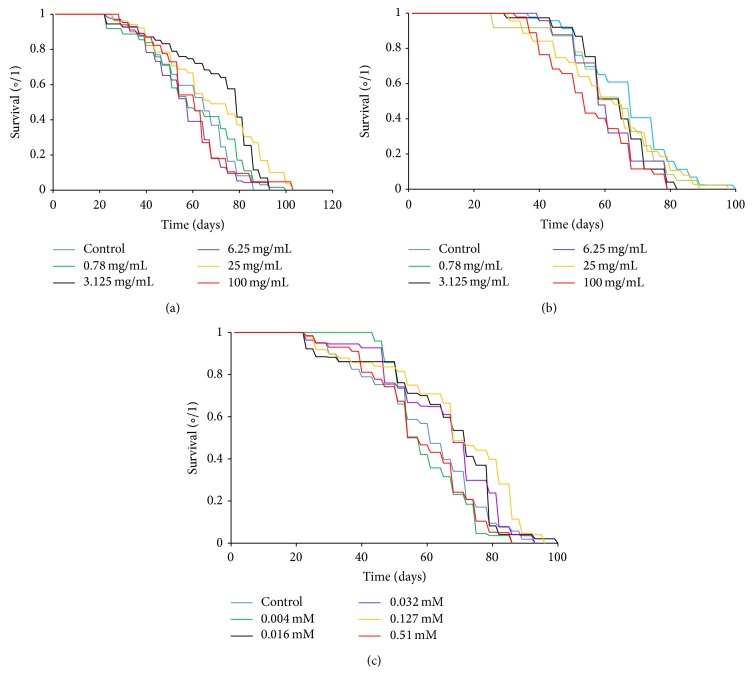
Effect of CCC (a), CFCC (b), and CAF (c) supplementation on the lifespan of* Drosophila melanogaster*.

Kaplan-Meier curves and averages of flies' lifespan are shown in Figure 1 and Table 4, respectively. The longevity of flies was increased by the CCC tested concentrations 3.125 and 25 mg/mL (*p* ≤ 0.05). CAF also increased the survival rates of* Drosophila* at intermediate concentrations (0.032 and 0.127 mM). CFCC significantly decreased the lifespan of* Drosophila* only at 100 mg/mL (*p* ≤ 0.001). On average whereas CCC and CAF increased* Drosophila* lifespan more than 15%, CFCC decreased it less than 19%.

Healthspan results (portion ≥ 80% of lifespan curves) are shown in Table 4. CCC increased the average healthspan of flies; such increase was significant only at 100 mg/mL (*p* ≤ 0.05) since this concentration raised the mean value by 22.4% to the control. Conversely, CFCC only significantly increased the mean healthspan value at 6.25 mg/mL (12%; *p* ≤ 0.05). CAF increased healthspan at the lowest (0.004 mM for 55.73%; *p* ≤ 0.01), the intermediate (0.032 mM for 40.32%; *p* ≤ 0.05), and the highest (0.51 mM for 26.41%; *p* ≤ 0.05) concentration.

### 3.2. *In Vitro* Assays

#### 3.2.1. Cytotoxicity

**Figure 2:**
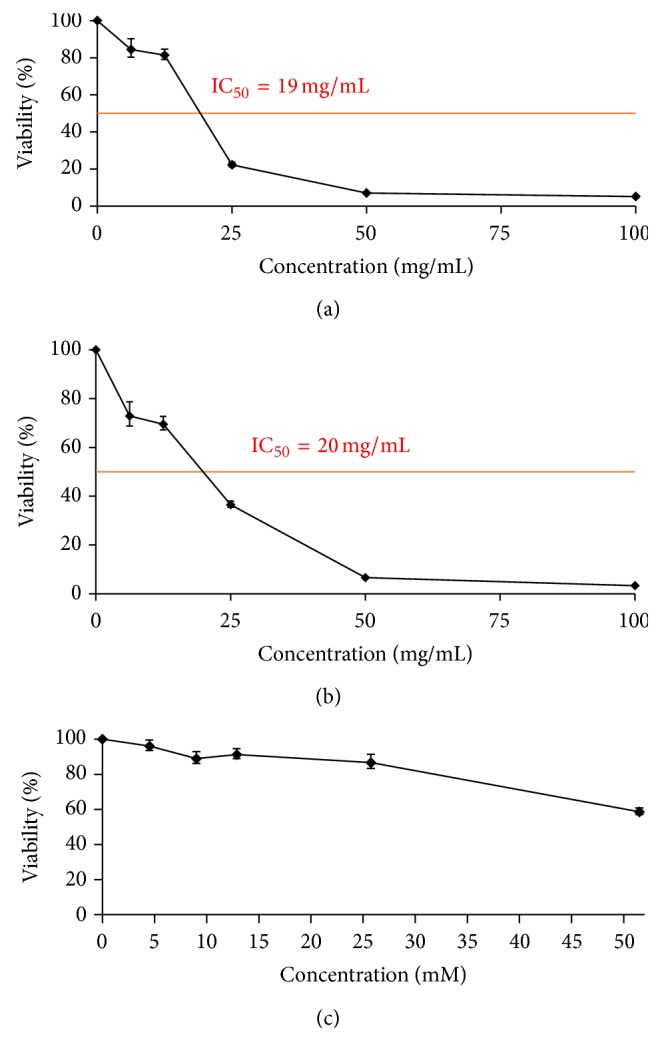
Cytotoxic effects of CCC (a), CFCC (b), and CAF (c). Viability curves at 72 h of treatment.

Both beverages were cytotoxic to the HL-60 line, inhibiting leukaemia cell growth with a positive dose effect (Figure 2). Furthermore, IC_50_ was similar for both beverages (19 and 20 mg/mL for CCC and CFCC, resp.). CAF concentrations were experimentally increased to reach IC_50_ since the original tested concentrations did not induce any remarkable cytotoxic effect on promyelocytic cells (data not shown). The highest tested concentration (20.4 mM), which was 40 times higher than the corresponding content in CCC and CFCC, could only inhibit cell growth in about 40%, without reaching IC_50_.

#### 3.2.2. DNA Stability Evaluation

**Figure 3:**
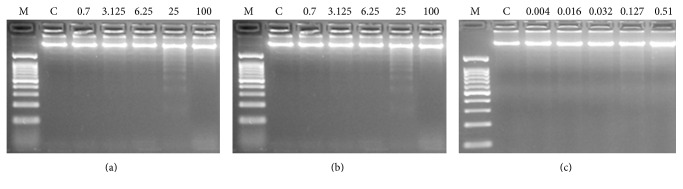
Internucleosomal DNA fragmentation after 5 h of treatment with CCC ((a)-mg/mL), CFCC ((b)-mg/mL), and CAF ((c)-mM). Letters M and C mean weight size marker and negative control, respectively.

The typical ladder pattern of cells with fragmented internucleosomal DNA was weakly induced only by CCC and CFCC at 25 mg/mL supplementation (Figure 3) and it was not observed with any CAF treatment.

**Figure 4:**
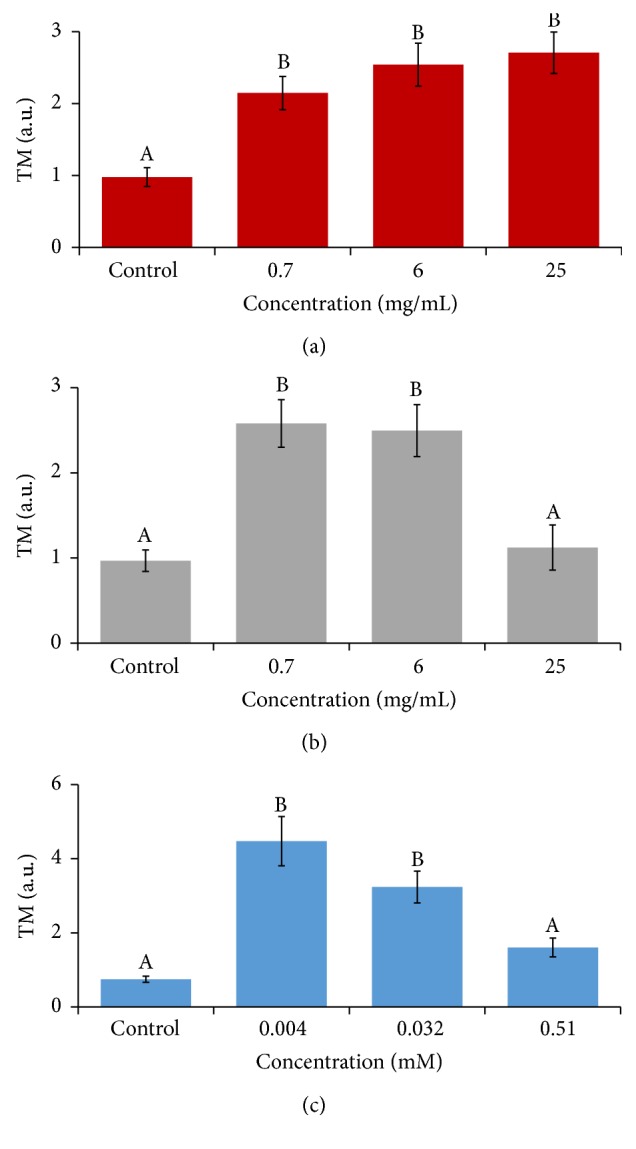
Alkaline comet assay (pH < 13) of HL-60 cells after 5 h treatment with different concentrations of CCC (a), CFCC (b), and CAF (c). DNA migration is reported as mean TM. The plot shows mean TM values and standard errors. Different letters mean different values after one-way ANOVA and* post hoc* Tukey's test.

The ability of the compounds to induce strand breaks in the DNA structure was determined by the alkaline comet assay. Based on the results obtained with the previous* in vitro* assays (cytotoxicity and DNA internucleosomal fragmentation), only three concentrations of each compound were tested. After 5 h exposure, all compounds induced a significant (*p* ≤ 0.001) increase in the TM parameter with respect to the control, except for CFCC at a 25 mg/mL concentration and CAF at 0.51 mM (Figure 4). Despite such significant increase, all TM values were lower than 4.4, suggesting that these compounds mainly affect HL-60 cells through a necrotic pathway.

**Figure 5:**
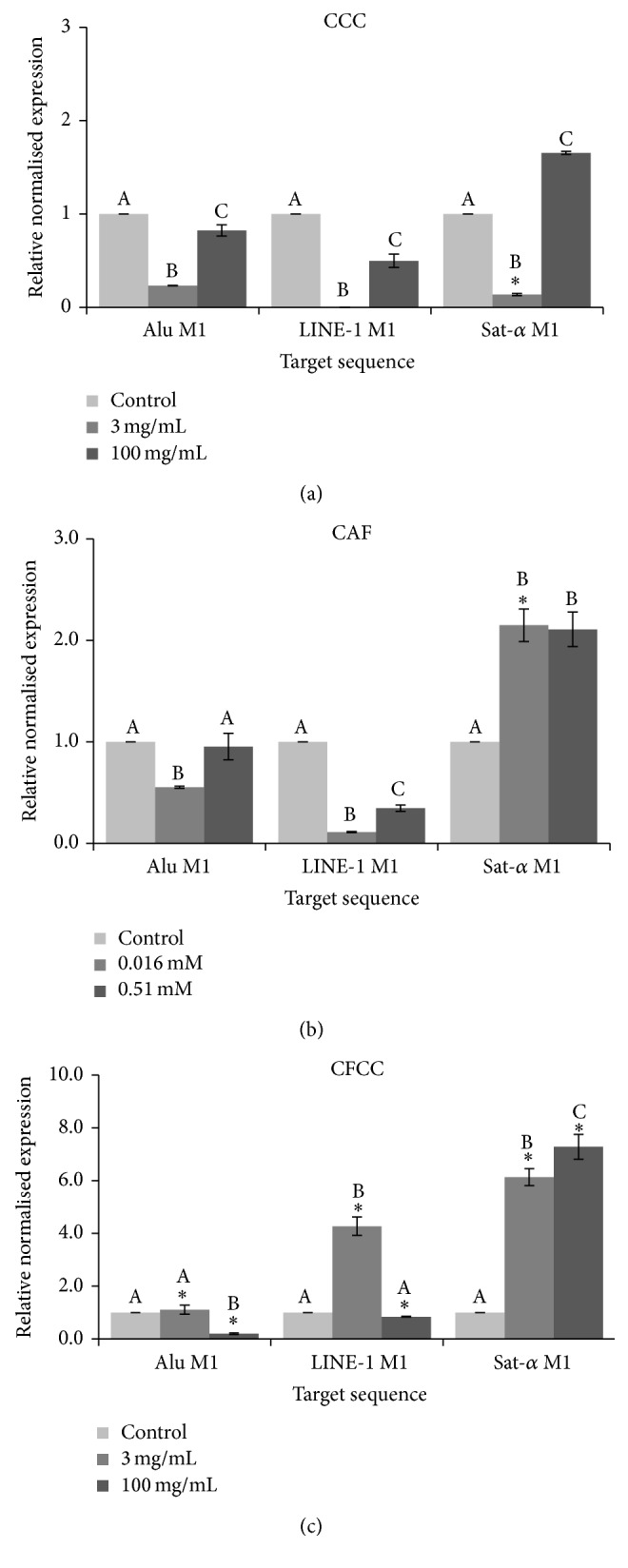
Relative normalised expression data of each repetitive element. Different letters are related to different means. Asterisks indicate differences among the same concentrations of the same substance for the different studied sequences.

The relative normalised methylation status (RMS) of the three repetitive sequences (LINE-1, Alu, and Sat-*α*) in HL-60 cell line treated with the tested compounds is shown in Figure 5. RMS decreased when cells were treated with CCC in both Alu M1 and LINE-1 sequences in a negative dose-dependent manner. However, we obtained hypomethylation in Sat-*α* sequences treated with 3 mg/mL and hypermethylation at the highest concentration (100 mg/mL) of CCC. CFCC induced hypermethylation in LINE-1 at 3 mg/mL concentration and hypomethylation at 100 mg/mL. A decrease of methylation status was found in Alu M1 sequences when cells were treated with 100 mg/mL CFCC. On the contrary, both assayed concentrations of CFCC were able to hypermethylate Sat-*α* sequences. Regarding CAF, a decrease of methylation status in Alu M1 and LINE-1 repetitive elements treated with 0.016 mM CAF and 0.016 and 0.51 mM, respectively, was observed. In contrast, an increase of the methylation status was found in Sat-*α* sequences when cells were treated with 0.016 mM CAF. The same demethylation pattern was observed at the three repetitive elements when looking at the same concentration as Tukey's test demonstrated when cells are treated with CCC and CAF, except for the lowest concentration of CAF when Sat-*α* is analysed. Nevertheless, CFCC differs from CCC and CAF as indicated by asterisks in Figure 5.

## 4. Discussion

### 4.1. Effect of Cola Beverages and Caffeine on* D. melanogaster In Vivo* Model

Soft drinks have been related to several harmful effects on health, such as child obesity and appetite increase, diabetes, hypertension, and dental diseases [6–8]. They were even related to school intoxication outbreaks, although in the end these events were associated with a mass sociogenic illness [49]. Nevertheless, studies assessing systematically the toxicological effects of cola beverages are scarce [50, 51] or showed contradictory results, as in the case of CAF.* Drosophila* is considered an accurate* in vivo* model to study human disease and further substantial contributions in this sense are expected [52].

To our knowledge, this is the first attempt to characterise the genotoxic effect of these beverages using* in vivo* (*Drosophila melanogaster*) and* in vitro* (HL-60) models, as well as CAF, using experimental doses mimicking the concentration used in cokes.

The lack of toxicity observed in our results is reasonable since these beverages are consumed worldwide and strictly regulated by governments and agencies. Furthermore, the use of “physiological” CAF doses could explain the harmlessness of the compound, since its effect was widely demonstrated as highly dependent of the dose consumed [53]. On the other hand, differences in sugar content between beverages (11.1% versus 10.6% W/V in CFCC and CCC, resp.) could explain the different toxicity levels found in the* Drosophila* assays. Several toxic and side effects were reported due to the high carbohydrate concentrations of beverages, particularly referred to as glucose and fructose. In our flies, it was also demonstrated that those carbohydrates could be converted into glyoxal which reduces the number of adults emerged and the pupation time [54].

In our study, only CCC and CFCC exerted a significant antitoxic activity against H_2_O_2_-induced oxidative damage in* Drosophila*. On the contrary, CAF showed neither toxic nor antitoxic effects. Since the effect of CAF has been widely described as dose-dependent, the lack of toxicity observed in our experiments was probably due to the low concentrations (equal to those found in the cola beverages) tested. In this sense, it was demonstrated that CAF can exert an antioxidant effect when consumed at moderate doses; it can even be neurotoxic at higher doses by increasing dopamine release [55, 56] or even inhibit autophagy in a dose-dependent manner [57]. Our results are more in agreement with Zhao et al. [58] who very recently found that CAF antioxidant properties are very weak and probably overestimated. On the other hand, it is well known that there are several extra compounds in Coca Cola, such as carbohydrate syrups, phosphoric acid (E-338), and class IV caramel colorants, but none of them has been reported as antioxidant [54, 59]. Therefore, we hypothesise that the antioxidant effects of CCC and CFCC could be explained by other undeclared components of these beverages, considering that part of its formula is an industrial secret.

Research using* Drosophila* has provided seminal insights into gene function which are relevant to human health [60]. The genomic stability (lack of genotoxicity) observed in* Drosophila* with all the compounds assayed confirmed their safety. Previous reports determined that cola drinks could be mutagenic by inducing chromosomal abnormalities and liver adducts in mice [61, 62]. However, those results are at least controversial, since the mutagenic effects were observed after 1 day of treatment with cola intakes equal to 600 mL in humans. On the contrary, our study agrees with Tóthová et al. [63] which demosntrated in a 6-month experimental design with rats drinking cola beverages* ad libitum* neither harmful effects nor changes in the gene expression pattern.

CAF is one of the most investigated genotoxic substances, probably because results obtained over time are not consistent (reviewed by Nehlig and Debry [64]). The absence of genotoxicity was reported a long time ago using different models: in* Drosophila* germ cells [65], in the* Salmonella* Ara test [66], or in the micronucleus assay [11]. On the contrary, mutagenic results have been reported after Sex-Linked Recessive Lethal (SLRL) test of* Drosophila* germ cells [67, 68]. Furthermore, it was demonstrated that CAF can enhance the effect of many DNA damaging agents [64]. Our results agree with those reported by Graf and Würgler [10], using the same experimental model. These authors demonstrated that CAF genotoxic effects are weak and nonsignificant.

An interesting finding was the antigenotoxic differences among both cola beverages and CAF. Our hypothesis is that the beverages effects could be mediated in part by the differential CAF content. Although* in vitro* studies indicated that CAF was able to scavenge hydroxyl radicals [69], this ability was not clearly observed in the highest concentration of our* in vivo* antigenotoxicity assays. In this sense, 0.51 mM CAF was not able to induce antigenotoxic activity although, contrarily, the lowest CAF concentration (0.016 mM) did induce it, being the most antimutagenic compound according to the recombination percentage data. In contrast, CAF has been demonstrated to be nonantimutagenic in Ames test at 0.19 mM [70] although it depends on the environmental factors [64]. Both cola beverages also revealed an inhibitory effect against the frequency of mutant spots induced by hydrogen peroxide due to an antimutagenic activity [71]. The different IP values of 166.67% and 96.93% for CCC and CFCC, respectively, at the lowest tested concentration could be due to the CAF content in CCC (0.016 mM CAF) since CFCC does not consist of CAF. This is in agreement with several reports showing CAF antigenotoxic capacity against X-rays [72, 73] and ethyl methanesulfonate (SMART assay [74] and yeast (15 mM) [75]). The IP value of CCC at 100 mg/mL decreased up to 98.88% and this fact could be due to the absence of antigenotoxicity observed in the highest CAF concentration. CAF did not present antigenotoxic activity in the micronucleus test of mice [11], although these authors assayed higher concentrations than those tested herein. However, CCC and CFCC antigenotoxic ability could also be due to another undeclared compound in the beverage formula or due to the presence of fructose, reported as being demutagenic against heterocyclic amines (Trp-p-1) [76].

*Drosophila melanogaster* is an excellent model for the study of aging because adults show many similarities with the cellular senescence observed in mammals [77]. This is the reason why this particular model is frequently used to understand the relationship between nutrient metabolism and aging mechanisms [25]. To our knowledge, the antiageing and antidegenerative effects of CCC and CFCC were assayed for the first time using* D. melanogaster* in our study. We demonstrated that CCC increased both lifespan and healthspan, whereas CFCC in general decreased both longevity indexes. However, these effects may not be related to the lack of mutagenicity produced by CCC and CCFC since there were no differences between beverages in the genotoxicity assays. Environmental factors, such as the diet of larvae, play a vital role in life expectancy. This was also reported in humans, associating sugared soft drinks with diabetes and obesity, both diseases playing an important role in the life expectancy decrease [78]. Therefore, the higher carbohydrate content of CFCC (compared with CCC) could explain the differences observed in longevity assays. We also demonstrated that CAF at 0.032 and 0.127 mM significantly increased lifespan in* Drosophila*, without having significant effects at lower doses. Interestingly, our results showed a reduced, even though not significant, lifespan in flies when higher concentrations were assayed. This was previously reported by Nikitin et al. [13], who demonstrated a negative effect of CAF in* Drosophila* lifespan with higher concentrations (25-fold higher than ours). A possible explanation could be that CAF produced a slimming activity by metabolism stimulation, associated with shorter life expectancies [79, 80].

### 4.2. Effect of Cola Beverages and Caffeine on* In Vitro* Cancer Model Cells

The* in vitro* evaluation of the anticancer properties of nutraceutical compounds or foods is the first step of a large pathway to obtain suitable conclusions to be extrapolated to humans. Here, we determined the potential chemopreventive effect of CCC, CFCC, and CAF on a human cancer cell model (HL-60 cell line). CCC and CFCC similarly decreased the survival rate of HL-60 leukaemia cells in a positive dose-dependent manner. Kapicioğlu et al. [81] reported the ability of cola drinks to inhibit proliferation of gastric mucosal cells although they were not cancerous. Conversely, Nowacki et al. [82] reported that CCC was able to induce an increase in fibroblast proliferation probably due to the sugar content, which could trigger a carcinogenic process. However, the rate of increase of this proliferation depended on where the CCC was bought. Our results showed that CAF induced weak cytotoxicity in HL-60 since none of the tested concentrations reached IC_50_. Therefore, we demonstrated that CCC and CFCC cytotoxicity cannot uniquely be due to CAF content. Previous reports showed that CAF inhibited HL-60 growth at 5 mM [83]. More recently, Rosendahl et al. [84] demonstrated an inhibitory effect of CAF against human breast cancer cells, IC_50_ being roughly at 5 mM. Similarly, Pitaksalee et al. [57] showed inhibition of autophagy with CAF supplementations of 10 mM in a neuroblastoma cell line. These recent reports support our findings, suggesting that CAF could be cytotoxic only at higher concentrations and in a positive dose-dependent manner.

The degradation of genomic DNA into internucleosomal fragments was proposed as a major mechanism affecting cancer cell apoptosis. We determined that CCC and CFCC only induced a weak proapoptotic DNA internucleosomal fragmentation at higher concentrations. Conversely, this activity was not observed in the concurrent CAF concentration tested. In this sense, previous reports by different authors are contradictory. It has been demonstrated that CAF protects HL-60 [14] and endothelial [85] cells against certain types of induced apoptosis in a dose-dependent manner and only at higher concentrations. The existence of a dose-dependent response pattern [55, 56] has recently been demonstrated by Wang et al. [86] showing that 2 mM CAF enhanced the proapoptotic effect of cisplatin lung cancer cells; these results could also explain the differences in CAF studies since they suggest that low CAF concentrations do not induce apoptosis by themselves, but by enhancing a different apoptotic pathway.

For these reasons, we performed alkaline SCGE in order to detect DNA damage [87], which are widely used to determine whether cells are undergoing apoptotic and/or necrotic pathways [41]. The use of such a test in transformed cells for the screening of substances with clastogenic DNA-strand break activity could be considered as a very early stage screening in the search of molecules for the treatment of acute promyelocytic leukaemia [88]. It is assumed that apoptosis occurs when treatments induce a TM > 30 (hedgehog pattern) whereas control cells remain lower than 2 (no tails). On the contrary, necrosis shows a short comet-tail pattern since the majority of the damaged DNA remains in the comet head [89]. Our results showed that the damage induced by CCC, CFCC, and CAF in HL-60 cells was characterised by necrosis (short tails, TM < 5, Figure 4). These results agree with our cytotoxicity and DNA fragmentation assays, demonstrating that CCC and CFCC induced cell death in HL-60, probably mediated by a necrotic pathway. Both beverages and CAF had the same DNA damage pattern (class 1; TM between 1 and 5 according to Fabiani et al. [90]) whereas class 0 was detected in their concurrent controls (TM lower than 1, no visible comet). In the same way, our results agree with those of Rayburn et al. [91] who reported that CAF supplementation (0–2 mM) did not produce DNA-strand breaks in CHO cells. Consistent with our results, several authors demonstrated that CAF induced apoptotic cell death in glioma and lung cancer cells at higher doses (10–20 mM), suggesting again that CAF acts in a positive dose-dependent manner [92]. However, recent studies demonstrated that CAF could induce a comet-tail pattern even at low concentrations (0.1–2 mM; [12]), but these reports were performed in yeast or in a different cell line (K562). Therefore, this could also suggest that CAF induced apoptosis differs depending on the* in vitro* model employed. Another interesting point is that the SCGE assay was described as relatively insensitive since positive results (no scored comets) would not be found when the tested compounds are highly cytotoxic [93]. However, despite the fact that beverages were cytotoxic in our study, this cytotoxicity assay was performed after 72 h of treatment and SCGE assays were conducted only for 5 h.

Regarding epigenetics, it is currently known that environmental factors are involved in gene expression. In cancer cells, the genome is globally hypomethylated inducing transposable element activity and thus triggering genome instability [94]. As a proof of that, the silencing of tumor suppressor genes is closely associated with hypermethylation [95]. Repetitive elements are highly methylated in somatic normal cells contributing to a global genomic hypermethylation [43, 94] suppressing the transposable activity of repetitive elements. Nevertheless, a lot of information is still unknown specially in order to ascertain the mechanisms which modulate the epigenetic changes in cancer cells. Biomedical research is focused on hypomethylation agents since this therapy is highly related to gene silencing; thus this fact could activate tumor suppressor genes and be a positive highlight although its benefit on human therapies is not clear because much more investigations should be performed [96].

We studied three different repetitive elements: LINE-1, Alu M4, and SAT-*α*. Long interspersed nuclear elements (LINE) are abundant retrotransposons and represent about 17% of the human genome. Although LINE1 has a nonrandom distribution, they are accumulated in primarily G-positive bands, which are AT-rich regions of chromosomes [97]. LINE-1 elements are also accumulated in regions of low recombination rate mainly in X-chromosome [98]. Alu elements belong to the SINE (short interspersed nuclear elements) family, being the most abundant (accounting about 10% of the whole human genome [43]) and predominantly present in noncoding and GC-rich regions [97, 99]. Sat-*α* (satellite alpha DNA) repeats are composed of tandem repeats of 170 bp DNA sequences, are AT-rich regions, and represent the main DNA component of every human centromere, constituting about 5% of total human DNA [97, 100]. Therefore, examination of the methylation status of LINE-1 and Alu regions has served as an approach for measuring global methylation levels since 32% of the human genome has been evaluated [101].

Our results of methylation status showed that CCC may generally hypomethylate the global genome although 100 mg/mL CCC hypermethylate Sat-*α* repetitive element. We also observed a significant negative dose-dependent effect in every target repetitive element with 50% hypomethylation average rate. Nevertheless, the overall hypermethylation rate induced in CFCC treatments is 328%, and only a decrease of methylation status is observed at Alu M1 and LINE-1 sequences when treated with CFCC 100 mg/mL. This hypermethylation could be considered as a benefit since LINE-1 is associated with C-met oncogene that would be silenced [102].

Xu et al. [103] demonstrated that caffeine (0.3 mM) enhanced the methylation ratio of multiple single CpG sites, as well as the total methylation ratio at nt −358 to −77 of the hippocampal 11*β*-HSD-2 promoter of primary fetal hippocampal neurons in rats. However, 4 and 40 *μ*M CAF were able to induce hypomethylation of single CpG site inhibiting the DNMT3 enzyme but not decrease the global status of the proximal promoter of the human StAR gene [104]. The present results of CAF are in agreement with Ting et al. [105] since 16 *μ*M was able to induce hipomethylation of Line-1 and Alu M1 sequences as well as 0.51 mM CAF in LINE-1. However, Sat-*α* (AT-rich elements) was methylated when cells were treated with 16 *µ*M CAF. It has been demonstrated that the expression of satellite sequences is associated with a hypomethylation triggering cancer cells; thus methylation process in satellite sequences is a potential mechanism for silencing its satellite expression in transformed cells [105]. These results could suggest that CAF may be one of the compounds responsible for the global hypomethylation status induced by CCC.

Statistical analysis showed that the methylation status induced by CCC and CAF in each repetitive element was not significantly different. Conversely, CFCC resulted in inducing different methylation status. Therefore, the effects of CCC on methylation status of HL-60 cells could be explained by those induced by CAF.

It is clear that much more information is needed for ascertaining on the role of food and beverages on epigenomes since hypomethylation mechanisms are not clear in every type of tumor. In addition, the hypomethylation and hypermethylation status of repetitive elements depend on both their concurrent control [102] and the target repetitive elements selected to evaluate the global methylation status. To our knowledge, it is the first attempt assessing DNA methylation changes induced by CCC, CFCC, and CAF on human leukaemia cells.

An apparent scarce data on the lack of dose-dependent effect is observed at almost all parameters analysed at the individual, cell, and molecular levels. Based on the obtained results, we only found a clear-cut dose-dependent effect when CCC is tested in the antitoxicity, cytotoxicity, and methylation bioassays. A threshold level of concentration may be needed to obtain some biological effects [106]. We found this threshold in the rest of the assays and compounds for toxicity, antitoxicity, longevity, healthspan, DNA fragmentation, and SCGE.

## 5. Conclusions

In conclusion, our experimental results show a slight chemopreventive effect of the two cola beverages against HL-60 leukaemia cells, probably mediated by nonapoptotic mechanisms. CCC and CAF induce a global genome hypomethylation evaluated in LINE-1 and Alu M1.

## Figures and Tables

**Table 1:** Primers information [43].

Primer	Forward primer sequence 5′ to 3′ (N)	Reverse primer sequence 5′ to 3′ (N)
ALU-C4	GGTTAGGTATAGTGGTTTATATTTGTAATTTTAGTA (-36)	ATTAACTAAACTAATCTTAAACTCCTAACCTCA (-33)
ALU-M1	ATTATGTTAGTTAGGATGGTTTCGATTTT (-29)	CAATCGACCGAACGCGA (-17)
LINE-1-M1	GGACGTATTTGGAAAATCGGG (-21)	AATCTCGCGATACGCCGTT (-19)
SAT-*α*-M1	TGATGGAGTATTTTTAAAATATACGTTTTGTAGT (-34)	AATTCTAAAAATATTCCTCTTCAATTACGTAAA (-33)

**Table 2:** Toxicity and antitoxicity levels of CCC, CFCC, and CAF in *D. melanogaster*.

CCC (mg/mL)	Survival (%)		CFCC (mg/mL)	Survival (%)		CAF (mM)	Survival (%)	
	Simple treatment^(1)^	Combined treatment^(2)^		Simple treatment	Combined treatment		Simple treatment	Combined treatment
0	100	100	0	100	100	0	100	100
H_2_O_2_	—	46.66	H_2_O_2_	—	46.66	H_2_O_2_	—	46.66
0.7	100	100^*∗*(3)^	0.7	87.66	83.33^*∗*^	0.004	100	54^Δ^
3	100	92^*∗*^	3	88.66	100^*∗*^	0.016	100	55.33^Δ^
6	100	85.66^*∗*Δ^	6	96.66	84.66^*∗*^	0.032	100	51^Δ^
25	100	74.66^*∗*Δ^	25	87.33	75^*∗*Δ^	0.127	100	54.66^Δ^
100	92	65^*∗*Δ^	100	77^*∗*(4)^	45.66^Δ^	0.51	100	51.33^Δ^

^(1)^Data are expressed as percentage of survival adults with respect to 300 untreated 72-hour-old larvae from three independent experiments. ^(2)^Combined treatments using standard medium and 0.15 M hydrogen peroxide. ^(3)^Asterisks (*∗*) indicate significant differences (one tail) with respect to the hydrogen peroxide control group and ^(4)^untreated control group: ^*∗*^Chi-square value higher than 5.02 [35]. Delta letter (Δ) means significant differences between the same concentrations used in toxicity and antitoxicity assays comparing within the same treated substance.

**Table 3:** Genotoxicity and antigenotoxicity of CCC, CFCC, and CAF in the *Drosophila* wing spot test.

Compound	Clones per wings (number of spots)^(1)^					Frequency of clone formation per 10^5^ cells^(2)^		Recombination (%)^(3)^	IP (%)^(4)^
	Number of wings	Small single spots (1-2 cells) *m* = 2	Large simple spots (>2 cells) *m* = 5	Twin spots *m* = 5	Total spots *m* = 2	Observed	Control corrected		
H_2_O									
*mwh*/*flr* ^*3*^	80	0.25 (20)	0.013 (1)	0	0.263 (21)	1.078			
*mwh*/TM3^(5)^	80	0.04 (3)	0		0.04 (3)	0.17			
H_2_O_2_ (0.15 M)									
*mwh*/*flr* ^*3*^	80	0.313 (25)	0.088 (7)	0.038 (3)	0.438 (35)+	1.795	0.717	54.37	
*mwh*/TM3	80	0.188 (15)	0.013 (1)		0.20 (16)	0.819	0.286		

*Simple treatment (mwh/flr* ^*3*^)									
*CCC (mg/mL)*									
[3.125]	80	0.275 (22)	0.025 (2)	0	0.3 (24)−	1.23	0.152		
[100]	78	0.19 (15)	0.038 (3)	0.026 (2)	0.256 (20)−	1.05	−0.028		
*CFCC (mg/mL)*									
[3.125]	80	0.175 (14)	0.075 (6)	0	0.25 (20)−	1.025	−0.053		
[100]	80	0.225 (18)	0.075 (6)	0	0.3 (24)−	1.23	0.152		
*Caffeine (mM)*									
[0.016]	80	0.26 (21)	0.03 (3)	0	0.3 (24)−	1.23	0.152		
[0.51]	86	0.21 (18)	0.058 (5)	0.012 (1)	0.28 (24)−	1.148	0.07		

*Combined treatment (mwh/flr* ^*3*^)									
*CCC (mg/mL)*									
[3.125]	82	0.11 (9)	0.037 (3)	0	0.146 (12)^*∗*^	0.6	−0.478	74.6	166.67
[100]	83	0.217 (18)	0.048 (4)	0	0.265 (22)^*∗*^	1.086	0.008	69.8	98.88
*CFCC (mg/mL)*									
[3.125]	82	0.195 (16)	0.073 (6)	0	0.268 (22)^*∗*^	1.1	0.022	55.5	96.93
[100]	80	0.175 (14)	0.05 (4)	0	0.225 (18)^*∗*^	0.922	−0.156	64.4	121.76
*Caffeine (mM)*									
[0.016]	80	0.16 (13)	0.025 (2)	0	0.188 (15)^*∗*^	0.77	−0.308	89.6	142.96
[0.51]	80	0.325 (26)	0.125 (10)	0	0.45 (36)^Δ^	1.844	0.766		

*Combined treatment (mwh/TM3)*									
*CCC (mg/mL)*									
[3.125]	79	0.038 (3)	0		0.038 (3)^*∗*^	0.158	−0.35		
[100]	80	0.08 (6)	0		0.08 (6)^*∗*^	0.328	−0.21		
*CFCC (mg/mL)*									
[3.125]	82	0.12 (10)	0		0.12 (10)*β*	0.49	0.32		
[100]	80	0.08 (6)	0		0.08 (6)^*∗*^	0.328	0.158		
*Caffeine (mM)*									
[0.016]	82	0.02 (2)	0		0.02 (2)^*∗*^	0.08	−0.09		
[0.51]									

^(1)^Statistical diagnosis according to Frei and Würgler [44]: + (positive) and − (negative) versus negative control; *∗* (positive), Δ (negative), and *β* (inconclusive) versus respective positive control; *m*: multiplication factor. Kastenbaum-Bowman Test without Bonferroni correction; probability levels: *α* = *β* = 0.05. Number of spots in parentheses.

^(2)^Frequency of clone formation: clones/wings/24,400 cells.

^(3)^Recombination percentage is calculated according to Valadares et al. [38].

^(4)^Inhibition percentage values were included when appropriate.

^(5)^Balancers-heterozygous wings.

**Table 4:** Effects of CCC, CFCC, and CAF treatments on the *Drosophila melanogaster* mean lifespan and healthspan.

	Mean lifespan (days)	Mean lifespan difference (%)^a^	Healthspan (80th percentile) (days)	Healthspan difference (%)^a^
*CCC (mg/mL)*				
Control	59.68 ± 2.92	0	32.63 ± 1.49	0
0.78	59.7 ± 2.6	0.04	29.67 ± 2.28	−9.08
3.125	69.78 ± 2.82^*∗∗*^	16.93	37.73 ± 2.58	15.63
6.25	59.81 ± 2.58	0.23	37.30 ± 2.26	14.32
25	69.16 ± 3.39^*∗*^	15.90	34.48 ± 2.17	5.57
100	64.34 ± 3.77	7.82	39.95 ± 0.96^*∗*^	22.44

*CFCC (mg/mL)*				
Control	66.05 ± 2.17	0	46.30 ± 1.90	0
0.78	65.7 ± 3.23	−0.99	42.16 ± 3.42	−8.93
3.125	66.86 ± 2.03	1.01	39.00 ± 5.00	−15.77
6.25	59.55 ± 3.57	−9.84	52.05 ± 1.93^*∗*^	12.43
25	66.06 ± 2.7	1.0	42.30 ± 0.67	−8.64
100	54.71 ± 2.17^*∗∗∗*^	−18.17	38.27 ± 1.09	−17.35

*CAF (mM)*				
Control	58.84 ± 2.46	0	30.18 ± 1.15	0
0.004	62.88 ± 1.7	6.87	47 ± 2.65^*∗∗*^	55.73
0.016	64.34 ± 2.75	9.35	36.18 ± 3.57	19.87
0.032	68.86 ± 2.38^*∗∗*^	17.02	42.35 ± 3.57^*∗*^	40.32
0.127	70.91 ± 2.99^*∗∗∗*^	20.52	36.91 ± 3.22	22.23
0.51	61.14 ± 2.07	3.91	38.15 ± 2.2^*∗*^	26.41

^a^The difference was calculated by comparing treated flies with the concurrent water control. Positive numbers indicate lifespan increase and negative numbers indicate lifespan decrease. Data are expressed as mean value ± SE. ^*∗*^ *p* ≤ 0.05, ^*∗∗*^ *p* ≤ 0.01, and ^*∗∗∗*^ *p* ≤ 0.001 significances obtained with the log-rank (Mantel-Cox) test.
